# Listening to Athletes' Voices: National Team Athletes' Perspectives on Advancing Safe Sport in Canada

**DOI:** 10.3389/fspor.2022.840221

**Published:** 2022-03-30

**Authors:** Erin Willson, Gretchen Kerr, Anthony Battaglia, Ashley Stirling

**Affiliations:** Safe Sport Lab, Faculty of Kinesiology & Physical Education, University of Toronto, Toronto, ON, Canada

**Keywords:** maltreatment, abuse, bullying, hazing, education, athlete, sport

## Abstract

With increased media scrutiny, public awareness, and research on the prevalence of maltreatment experiences in sport, sport organizations have faced increased pressures to combat unsafe practices in sport. A consequence has been the emergence of the Safe Sport movement whereby organizations including the International Olympic Committee, Safe Sport International, US Center for SafeSport, Sport Canada, and others, have developed policies, initiatives, and education intended to create safer sport environments for all participants. Most of these policies have been implemented using a top-down approach, driven by government officials and sport leaders. However, if safe sport initiatives are to benefit athletes, consideration and incorporation of athletes' perspectives in the development and implementation of initiatives are imperative. The purpose of this study, therefore, was to examine athletes' perspectives on the challenges and recommendations to advancing safe sport. As part of a large-scale survey of current and retired Canadian National Team Athletes' experiences of maltreatment, open-ended questions were asked about athletes' recommendations and considerations for safe sport. Responses to these questions (*n* = 386) were analyzed using thematic analysis. According to the participants, barriers and challenges to safe sport included emphasizing performance excellence at-all-costs, normalization and complicity of harm, lack of attention to equity, diversity and inclusion, a culture of fear and silence, and a lack of trust in organizations to handle cases of harm. In an effort to advance safe sport, participants recommended prioritizing holistic athlete development, improving and strengthening accountability measures, implementing an independent 3rd party for disclosure, reporting and support, increased attention to equity, diversity and inclusion, stakeholder education, prohibition of sexual relations between athletes and those in positions of power and authority, and adoption of a broader perspective of harms and perpetrators. Findings are interpreted and critiqued in light of previous literature and recommendations for future research and practice are suggested.

## Introduction

Prominent media reports indicate that maltreatment occurs in amateur and professional sports worldwide, as illustrated by Larry Nassar's conviction of sexually assaulting hundreds of United States gymnasts (Murphy, 2018) and NHL player, Kyle Beach, reporting sexual assault by a former hockey coach (Dotson and Sterling, 2021). The World Health Organization (2020) defines maltreatment as ill-treatment that “results in actual or potential harm to [an individual's] health, survival, development or dignity in the context of a relationship of responsibility, trust or power” and can include neglect, physical, psychological, and/or sexual harm. Neglect is characterized by an omission of care (e.g., withholding food or nutrition, training with injuries) (Stirling, 2009). Physical harm can be contact or non-contact, such as being hit, punched, slapped, or forced to hold a position for a longer than necessary period (e.g., splits for gymnasts) (Stirling, 2009). Psychological harm has been defined as patterns of belittling, denigrating, scapegoating, threatening, scaring, discriminating, ridiculing or other non-physical forms of hostile or rejecting treatment (Biggeli et al., 2001), all of which are viewed as threats to normal development (Garbarino, 1978). Sexual harm incorporates touching and non-touching sexual interactions (Ryan and Lane, 1997). Prevalence studies conducted in Canada (Parent and Vaillancourt-Morel, 2021; Willson et al., 2021), the United States (U. S. Center for Safe Sport, 2020), Belgium and the Netherlands (Vertommen et al., 2016; Ohlert et al., 2021), and a global study by World Players Association (2021) have provided insight into the rates of various forms of maltreatment in sport. Overall, these studies indicated that psychological abuse is most frequently reported by athletes, with 38–72% of athletes reporting at least one experience, followed by sexual abuse (9–30% of athletes) and physical abuse (11–21% of athletes) (Vertommen et al., 2016; U. S. Center for Safe Sport, 2020; Ohlert et al., 2021; Parent and Vaillancourt-Morel, 2021; Willson et al., 2021; World Players Association, 2021). The variance of prevalence rates may be a result of several factors, including different measures, inconsistency in terminology used to define harm, and the characteristics of the samples examined (e.g., level of sport, age of participants) (Vertommen and Parent, 2020; Lang et al., 2021).

With increased awareness of maltreatment experiences in sport, sport organizations have faced increased pressures to combat unsafe practices. A consequence has been the emergence of the Safe Sport movement whereby organizations, including the International Olympic Committee (2020), Safe Sport International^1^, U. S. Center for Safe Sport (2020), and Sport Canada^2^ have developed initiatives intended to create safer sport environments for all participants. Specifically, in Canada, the country of interest in the current study, numerous safe sport initiatives have been implemented in the wake of media reports of athlete maltreatment, prevalence studies, and the government's commitment, specifically from the former Minister of Sport, Kirsty Duncan (Canadian Heritage, 2019). These initiatives included a formal declaration to address maltreatment (the Red Deer Declaration) (Canadian Intergovernmental Conference Secretariat, 2019), implementation of a Canadian sport helpline for reporting maltreatment experiences in sport^3^, a baseline prevalence study of all forms of harm (Willson et al., 2021), the creation of a Universal Code of Conduct to Prevent and Address Maltreatment (see text footnote 3), an educational training module for all sport stakeholders (Coaching Association of Canada, 2021) and implementation of an Independent Safe Sport mechanism (Sport Dispute Resolution Centre of Canada, n.d.).^4^

Despite these advancements, Canadian athletes have reported dissatisfaction with the current safe sport landscape. For instance, in 2021, members of the Canadian National Women's rugby team claimed they had been let down by their sport organization's processes for handling maltreatment; specifically, “the process failed to protect [their team] and did not acknowledge the abuse and harassment we believe we suffered” (Davidson, 2021). Following the team's disappointment in the response from their sport organization, the rugby athletes used social media to garner attention using the hashtag “This Is Why Now” and a unified post describing their experiences. Bianca Farella, a rugby athlete who posted a statement using the hashtag, described the intent of the collective post as: “we've put out our statements, we've made our voices heard, and [it is now] out in the open” (Blum, 2021). Three-time Olympian Jennifer Heil shared her concerns about the current sport landscape: “the government knowingly allows the status quo to continue, which is policies without protection” (Karastens-Smith, 2021).

Many researchers in the Safe Sport field have generated research using athletes' voices to ensure engagement of those who experienced violence. Recently, the VOICE project comprised interviews with current and former European athletes with the intent of “listening to the voices of those who have been affected by sexual violence in sport” (Voice, 200). This project is based on the principle that “policy and initiatives generated from the accounts of those who have experienced sexual violence in sports settings will be highly valued, and therefore, most effective in ‘reaching' the sports community” (Voice, 200). Leaders of the VOICE project acknowledged that sport organizations do not always prioritize the rights and needs of athletes over their own reputations and believed their approach of integrating the athletes' voice was, therefore, unique and could produce meaningful change within the sport system (Brackenridge, 2002; Hartill et al., 2019).

The VOICES project aligns with a survivor-centered approach to violence, which has been defined by the United Nations (UN Women, 2013; GBV AoR, 2019) as “an approach that seeks to empower a survivor by prioritizing [their] rights, needs and wishes.” Kulkarni observed that one of the key components of a survivor-informed approach is the need for power-sharing between survivors and decision makers and that survivors should have a voice in all aspects of planning, delivery, and evaluation of services (Kulkarni, 2019). Survivor-defined practices should emphasize survivors' choices and autonomy, partnerships, sensitivity to the needs of individuals, provision of coping strategies, and opportunities for survivors to lead in the decision-making processes and voice their concerns (Goodman et al., 2016; Kulkarni, 2019). Kulkami highlighted that decision makers need to be reflexive of their own positions of power and privilege to avoid sustaining cycles of harm (Kulkarni, 2019). As suggested by Goodman et al. (2016), incorporating survivors' wants and needs into decision making and support resources can improve the empowerment of the survivors and their safety. Informed by research outside of sport, we suggest that a survivor-centered approach that embraces athletes' perspectives on safe sport initiatives could empower athletes and better ensure their needs regarding safety are met.

Despite the importance of genuine engagement with athletes when exploring maltreatment, including “the appropriate acknowledgment of lived experiences and the fostering of learning within sport organizations based on their testimony and expertise” (Hartill et al., 2019, p. 4), most of the safe sport initiatives in Canada have been implemented from a top-down approach, driven by government officials and sport leaders^2^ (Canadian Heritage, 2019; Canadian Intergovernmental Conference Secretariat, 2019). We propose that athletes' voices and perspectives must be elicited and incorporated in the development and implementation of safe sport initiatives if athlete welfare is to be advanced.

## Purpose

The purpose of this study, therefore, was to elicit athletes' perspectives of the challenges and recommendations to advancing safe sport.

## Methods

This study was conducted as a component of a larger research project exploring the maltreatment experiences of Canadian National Team athletes, including para and non-para-athletes. Collectively, 997 participants completed a comprehensive survey including closed, quantitative questions as well as two open-ended questions. The quantitative data on the prevalence, forms, and perpetrators of maltreatment have been published previously (Willson et al., 2021). The current study focused exclusively on the qualitative data retrieved from the open-ended questions of the survey pertaining to the challenges and recommendations for safe sport (see Participant and Data Collection Sections for detail).

### Research Paradigm

Given the focus of the current study was on exploring the data pertaining to athletes' perspectives on the challenges and recommendations to advancing safe sport, a social constructivist paradigmatic position was adopted. Within a social constructivist paradigm, emphasis is on unearthing the complexities of participants' subjective responses as well as the critical role of the researcher (Sparkes and Smith, 2014). Ontologically, a constructivist paradigm adopts a relativist perspective, which recognizes there are numerous views of reality that differ between participants (Sparkes and Smith, 2014). Epistemologically, a constructivist approach embraces subjectivist/transactional view, with the researcher and the participants actively involved in the co-construction of knowledge (Sparkes and Smith, 2014). A social constructivist paradigmatic position was appropriate for this study becuase athletes were invited to share their views regarding challenges and recommendations for advancing Safe Sport and we acknowledged our role as researchers in the interpretation of the data.

### Participants

While 997 athletes completed the survey, 265 participants (28%) responded to the open-ended questions. Participants who completed these questions were current and retired athletes, with retired athletes being defined as those who left sport in the last 10 years. Of the 265 respondents to the open-ended questions, 56.2% (*n* = 149) were current athletes and 43.8% (*n* = 116) were retired athletes. Thirty-two percent (31.7%; *n* = 84) identified as men, 67.5% (*n* = 179) identified as women and one (*n* = 1) participant identified as neither man or woman. The mean age was 31.7 years. Athletes with a disability constituted 10.2% (*n* = 27) of participants; racialized athletes constituted 10.9% (*n* = 29) of participants; 9% (*n* = 24) of participants identified as LGBTQ2I+; and one athlete identified as Indigenous. In the Canadian context, the label “Indigenous” is understood as Canadian First Nations, Inuit and Métis.

### Recruitment

Following ethical approval from the University Research Ethics Board, athletes were contacted through the AthletesCAN email listserv and social media posts (Facebook, Instagram, Twitter). AthletesCAN is the association for Canadian National Team Athletes, who partnered with the authors to refine and distribute the survey. Recruitment communication included a link to participation, a letter of information and consent with inclusion criteria, and links to the survey in French and English. The online survey was anonymous and completed surveys were routed directly to the research team. The survey was available online for 30 days, and two reminder emails were sent during this time. Compensation was not provided for participation. Due to the sensitive nature of this study, participation was voluntary, and participants were informed they could skip any questions they did not feel comfortable answering. The participating athletes were assured in the letter of information and consent that their answers were confidential and only aggregate and anonymized data would be presented.

### Data Collection

An anonymous, online survey was created for this research project (see Willson et al., 2021). The survey consisted of 121 questions that assessed several topics of interest, including identity characteristics (age, race, sport, gender, sexual orientation, Indigeneity, and para-status), athlete satisfaction, wellbeing, experiences of various forms of maltreatment, and experiences with disclosure and reporting. Concluding this survey were two open-ended questions asking about athletes' recommendations and considerations for safe sport; specifically, these questions included: (1) Is there anything else you would like to add that has not already been discussed? and; (2) Based on your experiences, do you have any recommendations to advance Safe Sport? Data retrieved from responses to these two questions were the focus of the current study. These questions provided an opportunity for athletes to elaborate on their perspectives and to express their recommendations for advancing safe sport.

### Data Analysis

The responses in French were translated to English by a bilingual graduate student and analyzed by the English-speaking research team. Due to the common overlap of responses between the two questions, for example, a respondent answering “The NSO [National Sport Organization] has ultimate power” for Question 1 and “a third party is needed for athletes to feel safe so they do not need to report to an NSO”, all the responses to both questions were combined for the analysis. In total, from the 275 participants who answered at least one of the two open-ended questions, there were 386 responses used in the analysis. The responses to the open-ended questions were analyzed using an inductive thematic analysis (Braun and Clarke, 2006; Braun et al., 2016), thus allowing categories and themes to be interpreted from the participants' responses, rather than being assigned into predetermined categories and driven by existing literature (Braun et al., 2016). Data retrieved through open-ended survey responses were then organized and interpreted into meaning units and overarching themes pertaining to challenges and recommendations to advancing safe sport (Braun et al., 2016).

### Qualitative Rigor

A relativist approach was adopted to ensure the quality of the research, which views criteria for rigor as a list of characterizing traits that vary depending on the context and purposes of the research (Sparkes and Smith, 2014; Smith and McGannon, 2018). The specific criteria that were used to assess rigor include significance of the research contribution (e.g., timing with rise in Safe Sport initiatives and novelty of the findings); credibility of data (rich and thick descriptions); ethical considerations (e.g., anonymity, providing participants with mental health resources, transparency in reporting their data); meaningful coherence (e.g., meaningful connections throughout the research questions, findings, and interpretations) and; naturalistic generalizability (i.e., the extent to which the research resonates with the readers' experiences) (Smith and McGannon, 2018). Considering the depth of information presented regarding athletes' views of the challenges and recommendations to advancing Safe Sport throughout the results, it is speculated that the responses may also resonate with other athletes and sport stakeholders more broadly.

### Ethical Concerns

In recognition of the sensitive nature of this topic, the ways in which various ethical concerns were addressed will be described. The letter of information detailed ethical considerations, the survey was anonymous, and participants were assigned a number for the dissemination of results. Any identifying information (sport, specific names of coaches or sport administrators, name of sport organizations) was removed. Following the completion of the survey, participants were provided a link to local mental health services and an option to contact AthletesCAN if more assistance was needed. The anonymity of the survey meant that the researchers could not enact a legal duty to report should a criminal act be revealed in the athletes' responses; this was identified in the letter of information.

## Results

The purpose of this study was to examine athletes' perspectives on the challenges associated with as well as recommendations for advancing safe sport. The athletes highlighted numerous challenges that reportedly hindered safe sport experiences, which included: normalization of harm and complicity; performance excellence at all costs; culture of fear and silence; and a lack of attention to equity, diversity and inclusion. Consistent with these challenges, athletes provided recommendations for advancing safe sport, such as prioritizing holistic athlete development, education for all stakeholders, prohibition of sexual relations and forced intimacy, and mandating independent complaint process and ensuring accountability. Each of these themes are described in the following section.

### Challenges for Advancing Safe Sport

#### Normalization of Harm and Complicity—“Horrible Conditions and Abuse Were Glorified”

Athletes expressed concerns about the acceptance or normalization of specific harmful behaviors in sport and suggested that key sport stakeholders knew of inappropriate or harmful practices but failed to act on their responsibilities to protect or ensure the welfare of athletes. A65 indicated that certain practices are not only normalized and harmful but viewed as necessary for performance outcomes, “The high-performance director thinks that the tougher the coach is the better we will become so horrible conditions and abuse were not just tolerated but in fact, glorified.” A172 commented:

There is still a culture of excusing inappropriate coach behavior by labeling them as “passionate.” Former and current coaches known to be physically and psychologically abusive toward children are being honored in our sports hall of fame… Parents are brainwashed into thinking that only the toughest coaches will turn their children into champions and support them blindly…they turn a blind eye to the unsafe training conditions of their own children.

According to the athletes, common normalized behaviors were psychologically and sexually harmful practices and body shaming. A185 stated:

With my coach, boys especially get harassed, humiliated, threatened, bullied on a regular basis. I am routinely told I am obese, fucking useless, I have no talent, my [skill] is dog shit, I eat wrong, we walk too loud—I could go on forever. Every day during team activity, I am told I can be replaced, I will be sent home, I am off the team if I don't “x”, there is a plane ticket waiting for me…I am not allowed to eat much, because I am constantly being told I am fat—I was hungry all the time while traveling this past season—my food was controlled so that I would lose weight. I get treated like this and I am the top male athlete on the team.

For some athletes, the acceptance and normalization of certain experiences was so engrained in their sport culture that it prevented them from labeling experiences as maltreatment. For example, A51 commented, “My current coach is amazing—no one is perfect, but with one exception—[being ignored] after poor results, which I think is just because he cares too much and isn't always tactful in his feedback.”

#### Performance Excellence At-All-Costs—“No Medal Will Ever Justify How I Was Treated”

Athletes referred to an emphasis on performance excellence throughout their sport participation; specifically, that their ability to win and consistently perform at a high level took precedence over their holistic development, thus limiting safe sport experiences. A172 explained, “Up and coming athletes are treated like pawns to the glory of the coach and high-performance director and are neglected, shamed, and damaged when the coach and NSO discover they are not perfect.” The emphasis on achieving performance excellence was also acknowledged in relation to funding implications:

I watched an entire generation of athletes between ages 16–27 be purged and kicked off the team since they were not “needed” and there was “no value” in development based on the funding model. A258

Athletes reported feeling they were worthless to the organization once they retired because they were no longer contributing to performance success. A83 stated, “Anxiety, depression, and PTSD [Post-Traumatic Stress Disorder] occur years after retirement due to improper retirement protocols and NSOs not giving a shit about you after you retire…they build you up…then when you don't make the top three, they forget your name.” Similarly, another athlete who also felt ignored after retirement recognized that tolerating abuse was the only way to succeed in their sport, and they added “… no medal justifies how we were treated. I will never get over it” (A258).

Athletes also recognized that harmful coach behaviors were tolerated in the pursuit of performance excellence. A258 described the attainment of performance goals serving as a protective mechanism against perpetrators of harm, “As long as people were achieving results, the coaches and high-performance directors could act with absolute immunity.” Another athlete (A50) echoed this sentiment: “Our team was successful, so they kept him on board despite the abuse.” Informed by the importance of satisfying performance expectations, athletes also described situations where they felt pressure to perform while injured. For example, A26 stated, “I felt pressure to compete when injured and fatigued. While it was never overtly expressed, the communication from my then high-performance director made it feel as though not competing wasn't a consideration.“

#### Culture of Fear and Silence—“I Am Fearful That After I Speak Out, I Will Be Punished”

A common sentiment expressed by the athletes was a reluctance to disclose or report accounts of unsafe sport experiences; specifically, athletes referenced a sport culture characterized by a fear of repercussions. A70 expressed a desire to speak up about their experiences but acknowledged the potential costs: “I should have done more, not just for myself but my fellow athletes but the fear of being blacklisted, kicked off a team, or losing funding kept me silent when I witnessed cases of bullying and harassment.” Further, A164 stated:

Knowing we can be replaced, and our careers are on the line, you are regularly forced to ignore issues or maltreatment out of fear. I have witnessed blackmail, intimidation, favoritism, and experienced verbal and mental abuse. We are silenced if we ask questions. I am fearful that after I speak out, I will be punished.

For some athletes, the repercussions for addressing safe sport issues were highlighted as A39 explained, “I did not retire; I was barred from competing for speaking out against my coach/NSO and kicked off the team. If I kept my mouth shut and tolerated abuse and discrimination, I would still be on the team.”

The athletes also identified procedural obstacles that further served to foster fear and silence their voices on matters of safe sport. A38 commented, “I did not make a formal complaint because the process was lengthy, and I was afraid of repercussions and mentally it would be difficult to deal with and continue training.” Likewise, A35 commented:

While NSOs have policies in place, for issues (neglect, favoritism, not following through on promises, etc.) that have no place in sport…there is nowhere to go—the athlete is trapped with no recourse. If you say anything to the coaches or admin, you are a troublemaker. If you even tried to go to the Board, they are all friends of the staff so they will deny your case.

Similarly, athletes have noted the imbalance of power that is created by an environment that is supportive of abusive coaching. A172 summarized:

Our sport is organized into clubs run by volunteer board of directors. Abusive coaches stack the board with the parents they want, then the club boards misdirect child services and pressure witnesses/bury abuse complaints under the rug. They also turn a blind eye to the unsafe training conditions of their own children.

The lack of trust athletes had in their sport organizations was a prominent theme for athletes, which they attributed to fostering negative experiences. Athletes described situations in which complaints were reported but little action was taken by Provincial Sport Organizations (PSO) or NSOs to address unsafe sport. A183 claimed, “When I reported an instance of sexual bullying and verbal harassment toward a teammate to the administration, I received zero support. No repercussions to the individuals were handed out and the behavior continued toward that teammate.” Referring to a harmful coach, A108 explained, “They recommended firing the coach. Over five players have left our program due to mental health issues in direct correlation with the head coach. Our team was successful, so they kept him despite the abuse.” Similarly, A10 commented:

As a senior athlete sitting on a PSO board, I was contacted by a U23 female athlete who had been sexually assaulted by her coach at a national team event. I went to the police station with the athlete to file an official complaint. I also reported the incident to the PSO. A few other athletes came forward with similar sexual misconduct complaints by that same coach. The police never did anything…the PSO swept the problem under the rug. To this day, that coach is still out there, coaching young female athletes.

#### Lack of Attention to Equity, Diversity, and Inclusion—“When Bringing up Diversity and Inclusion, I Was Met With Homophobic Remarks”

According to athletes, an impediment to safe sport has been the lack of attention of sport organizations and its stakeholders regarding equity, diversity, and inclusion and the needs and rights of vulnerable groups. A172 commented, “Clubs turn a blind eye and excuse bullying of young LGBT [athletes] by their peers as 'kids being kids.”' Likewise, A25 reflected:

In meetings with our NSOs High Performance Director and relevant administrators, they were not receptive to making changes that would promote a safer sport environment such as…releasing a statement in support of diversity and inclusion. In particular, when bringing up the topic of diversity and inclusion, I was met with homophobic remarks. There was a perception that such things were not the responsibility of the NSO.

The inability to acknowledge how sport experiences may differ for such groups as female athletes, racialized athletes, and parasport athletes, was also mentioned as hindering safe sport for all. Referencing disparities in gender equity, A65 commented, “Our team has been neglected by our high-performance director for over a decade. There is not enough adequate support to help women in sport right now—we let a lot of things slide that absolutely should not.” A10 noted the casual racial discrimination that can occur in sport “Awareness of race and the jokes/harassment/treatment that can be made at the expense of someone who is a visible minority within a sport.” Likewise, addressing prevalent issues in parasport, A73 stated:

The parasport community has to do a better job of tackling internalized ableism. So much in that community is about training as hard as able-bodied athletes, certain disabilities are routinely mocked, anyone whose disability contributes to inconsistent performance (fatigue/pain-based) is told that it's their fault and they need to get stronger mentally. A lot of my injuries were the result of training in a way that was not recommended for someone with my disability. Because it's a small community, toxic people who don't want their spot on the national team threatened can have a huge impact on bullying up-and-coming athletes.

### Recommendations for Advancing Safe Sport

#### Prioritization of Holistic Athlete Development—“We Need to Value Athletes as People First, Not Solely on Their Ability to Get Medals”

Athletes suggested that advancing safe sport requires a shift away from a singular focus on performance success at-all-costs and toward holistic athlete wellbeing. A166 commented, “The power in sport needs to be shifted back to the athletes. Athletes need to be seen as having value and not to be seen as a commodity that is easily replaced.” Likewise, A72 commented, “Sport culture needs to change to value the holistic development of athletes (mental, physical, emotional, and spiritual). That comes with placing less emphasis on winning and providing evidence-based education to increase literacy surrounding the wellbeing of athletes.” Associated with such values, athletes also expressed desires to be treated and viewed as more than just an athlete. A75 stated:

We need to value athletes as people first, not solely on their ability to get medals. To do this isn't to say winning doesn't matter, but it's to respect a fair and transparent selection process for everything at all times. This gives athletes agency, which can in turn lead to them feeling better about their sport journey regardless if they achieved their goals or not.

In order to foster positive athlete development, athletes suggested that current funding practices require reconsideration. A333 explained:

To truly advance safe sport Canada needs to take a long hard strategic look at how they are funding athletes as well. As long as the pressure and bottom line of money for medals exists, challenges around safety in sport will remain because the pressure to perform and the impacts of other people on athletes to perform will continue.

#### Education for All Stakeholders–“I Didn't Know the Way I Was Treated Was Inappropriate”

The athletes made recommendations for further education for coaches, athletes, sport administrators and high-performance directors. A332 suggested, “To educate administrators and high-performance officers so they are aware of the implicit cues athletes receive that make athletes feel pressured not to report things and keep quiet about unfair practices (fear of losing funding, support, team selections etc.)” A86 suggested, “If everyone can be aware of grooming and understand what abuse looks like, more people may step in and recognize or save an athlete from being abused.” Education pertaining to power-dynamics was highlighted as a specific area of importance:

One of the biggest tools we have is understanding power dynamics. I noticed a lot of athletes drawn to the attention of the coach and the coaches being excited to have so much power and influence. I had teammates competing for coaches' attention and it made a very unhealthy dynamic. A303

Prioritizing athlete education and training was also identified as a way in which to advance safe sport. A37 commented, “Athletes need to be educated to know their rights…I didn't realize that the way I was being treated was inappropriate.” With regards to improving knowledge of reporting and support services, A199 stated, “Athletes should be made aware of reporting procedures for all sports and the national federation should mandate this knowledge be part of national team induction events.”

The athletes highlighted the importance of setting educational qualification requirements for adult leaders who interact with athletes, most notably coaches. A72 argued:

More stringent requirements in becoming a coach…Too often coaches earn their positions from having been athletes themselves – that is absolutely no reason to think they are qualified to work with national team athletes. These requirements would also include evidence based educational intervention to not only inform coaches about all forms of abuse and mental health, but also their role in facilitating a sport environment that promotes help-seeking and minimizes the risk of such issues from arising... In areas where coaches are identified as being deficient, they should be offered additional support to enhance competencies.

Athletes also referenced the need for education to extend beyond a predominant focus on sexual harm to include other forms of harm. Athletes discussed specific examples of harm that are not often recognized such as: “[my sport] is a toxic environment of harassment, disrespect and utter discrimination”—A30; “My coaches were mentally abusive, and it's gone unnoticed”—A99; and “Harassment and bullying are very serious issues”—A186. Similarly, A331 stated:

There needs to be an EXPLICIT understanding of what abuse is—in its physical AND emotional AND mental realm…Coaches need to know when they belittle an athlete it's abuse; when they withhold things necessary in an athlete's life, it's abuse; when a technician is in control of equipment and is sleeping with some of the athletes it's abuse; when coaches are sleeping with athletes it's abuse of the athlete and all the others who have a right to be equally coached by that coach...You NEED to shine a light in the darkest of places by giving the behaviors a name: when a coach or anyone else who has power over you pressures you to do something you don't want to do.

Although many athletes referred to maltreatment perpetuated by coaches, they also emphasized the importance of attending to harm that arises between peers. A71 stated, “In addition to harassment and abuse from coaches we also need to shine a spotlight on abuse from teammates.” A160 suggested coaches learn how to prevent and intervene in cases of peer maltreatment:

Coaches should be required to go through training to help them navigate harassment and abuse issues between team members. My coach always put his head in the sand and said, ”that's not my job.” I know there is a focus on dealing with harassment and abuse perpetrated by coaches, but I think that issues between peers also have to be seriously addressed.

According to the athletes, advancing safe sport requires greater education with respect to needs and rights of equity-deserving groups. For example, A264 commented on the importance of coaches understanding how to work with female athletes in a safe manner, “Coaches of teenage girls need to be trained in how to work and communicate with females specifically.” Further, the importance of understanding the unique needs of able-bodied vs. non-able-bodied athletes and the impacts for their safe sport experiences was also mentioned. A72 stated:

It is important for research to be conducted into parasport specifically to assess the safe sport environment. I believe the types of violence and abuse are at a different level and people with disabilities are marginalized even more, so coming forward will likely be more challenging. Give proper attention to specific marginalized groups *via* further research and measures. Measures put in place cannot be a one size fits all. Addressing issues of able-bodied athletes vs. disabled athletes can be very unique, service providers need adequate training, and the athletes need to feel like the protocol and service is tailored to them in order to be effective.

#### Prohibition of Sexual Relations and Forced Intimacy—“Zero-Tolerance of Coach-Athlete Sexual Relationships”

When addressing forms of harm in sport, athletes specifically recommended prohibiting sexual relationships and forced intimacy between athletes and persons in positions of power and/or authority in sport. A89 stated, “Zero tolerance of coach-athlete sexual relationships. The power imbalance is too great to allow for consent.” Similarly, A284 argued, “Make it against the rules for people within the national team staff (e.g., coach and physiotherapist) to be involved in a romantic relationship.” The importance of addressing such concerns is apparent when considering the consequences athletes may endure when demonstrating an unwillingness to engage in inappropriate relations. A43 commented:

I experienced discrimination and was treated negatively while working with a male coach that enjoyed flirtation with female athletes. I maintained a clear coach-athlete relationship…I suffered a lack of feedback and attention because I didn't engage in flirtation with the coach.

#### Mandating Independent Complaint Processes and Ensuring Accountability—“if You let Them Run Their Own Investigations, You Let Them Cover Up Their Mistakes So They Don't Get in Trouble”

A widespread recommendation suggested by the athletes was the implementation of a third party, neutral, independent body where athletes can go when they feel they have faced maltreatment. Athletes referred to this independent body as being completely disconnected from their NSOs; a place where they can go to disclose an experience and receive support even if they choose not to submit a formal complaint as well as a place to submit a formal report and receive support throughout the investigative and adjudication processes. A121 stated, “If we are ever to truly have safe sport, an authoritative, confidential, and independent body must be put in place.”

According to the athletes, the independent body would specifically help to limit fears of repercussions, conduct regular audits to ensure actions and efforts toward athlete welfare, and diminish the likelihood of sport organizations and their stakeholders engaging in biased review processes. A270 stated, “I would never feel comfortable going to my National Sport Organization if I were harassed and would 100% need an individual body to report the harassment too. I would be far too scared.” Referring to annual audits, A276 described, “… an independent body that handles investigations and a requirement for National Sport Federations to be regularly audited by an independent body on their efforts and actions to change the culture of athlete welfare (policy, climate surveys, self-evaluations of sport, etc.).” Further, A58 explained:

A third party that the athletes can go to when they feel they have faced abuse, discrimination, harassment. This independent body is funded by Sport Canada and operates independently of NSOs…they file and review cases. Don't let the NSO tell you everything is fine or trust they will do the right thing. Telling NSOs about a concern means putting them in a position where they have to incriminate themselves…if you let them run their own investigations you let them cover up mistakes, so they don't get in trouble.

Athletes also highlighted the importance of ensuring accountability of NSOs, such as regulatory processes to hold NSOs accountable to adhering to appropriate policies, procedures, and practices. A72 indicated, “NSOs need to be held accountable and be regulated more closely to ensure they are following appropriate guidelines. Our high-performance director was given too much authority and created an unfair environment where selection was unethical and resulted in the emotional distress of athletes.” Athletes specifically articulated a need for NSOs to hold coaches accountable for their conduct.

When an athlete or team says that the coach is unfit and that her behavior is considered harassment, listen! It is not ok to “wait and see” what will happen and expect that all problems will resolve themselves. When 12 people give you different instances of unacceptable behaviors, that means there is a problem, don't tell your athletes that they are “just being dramatic and will have to deal with it.”—A75

Further, athletes recommended regular assessments of NSOs' actions to change the culture of athlete welfare through climate surveys and athletes' evaluations of their sport experiences. A208 suggested, “Ask for more direct feedback from athletes, bypassing sport governing organizations. Federal and provincial sport governing bodies do not solicit, share, or consider athletes' feedback.” Likewise, A126 commented:

Sport Canada and AthletesCAN could implement a survey every year to understand the athletes' perspective on the NSO—How well do you feel your NSO is managing funds? How objective/fair do you feel your NSO is in making decisions? Do you feel you can go to your NSO when you have an issue? Do you trust your coach/NSO, etc. with your best interests? These responses would give Sport Canada an idea of underlying issues that may need to be investigated or help with resolution.

## Discussion

The purpose of this study was to examine National team athletes' insights into the challenges and recommendations for advancing safe sport. Part of a larger project assessing the prevalence of maltreatment among National team athletes (Willson et al., 2021), the results conveyed in this article emerged from two open-ended questions that asked athletes about their recommendations for advancing Safe Sport and whether there was any other information they would like to add. The richness of responses to these open-ended questions was a unique finding, with almost 400 in-depth responses provided by athletes. Given the comprehensive responses that were provided by the athletes, we believe this study provided athletes with an avenue of empowerment, and a means to express their needs and wishes, which aligns with a survivor-centered approach to addressing violence (United Nations). This study adds to the existing literature by inviting athletes to comment on their perspectives of maltreatment of all forms and on a large scale.

According to the athletes, one of the prominent challenges to safe sport is a prioritization of performance excellence in Canadian sport. Athletes in the current study highlighted the almost singular focus on performance when noting that athletes were praised when they are succeeding and neglected or ignored when their performance did not meet expectations, if injuries occurred, or if athletes were unwell or suffering from mental health challenges. This is consistent with previous research on maltreatment, which has indicated that a win-at-all-cost mentality supersedes the health, wellbeing, and development of athletes (Jacobs et al., 2017; Wilinsky and McCabe, 2020). While performance outcomes are important in high performance sport, the singular focus on results creates rigid standards of what it means to be a good or successful athlete and, as a result, enduring harm in the pursuit of performance excellence becomes normative and necessary (Roberts et al., 2020). To address these challenges, the participants highlighted the importance of valuing holistic athlete development. Specifically, the athletes indicated that success should not be defined according to short-term outcomes (e.g., outperforming opponents, displaying mental toughness) but to long-term outcomes such as athletes' social, emotional, and cognitive development/wellbeing. The athletes in this study also identified the importance of sport and its stakeholders valuing and empowering athletes through their sport participation, which aligns with previously proposed solutions to maltreatment that focus on athlete-centered practices and holistic development (Miller and Kerr, 2002; Donnelly et al., 2016). Athlete-centered coaching has been defined as an approach that “promotes athlete learning through athlete ownership, responsibility, initiative and awareness” (Pill, 2017, p. 1). Through this style of coaching, athletes and coaches are in partnership, athletes' needs (including physical and emotional) are prioritized, and athletes contribute to the decisions that affect them. Benefits of an athlete-centered approach include increased confidence, perceptions of success, motivation, stronger connections with the coach, autonomy, team compatibility, and longevity in sport (Falcão et al., 2020; Pill, 2017; Falcão et al., 2019; Dohsten et al., 2020). In sum, the athletes in the current study recommended a shift in the culture of high-performance sport to forefront athlete health and wellbeing.

Interestingly, the calls to reduce the win-at-all-cost approach to sport have existed in Canada for decades. For instance, following the use of banned substances by Canadian runner Ben Johnson in 1988, the resulting investigation highlighted the over-emphasis of high performance as an underlying cause of athletes' decisions to use performance enhancing drugs (Montague, 2012). Specifically, the investigator, The Honorable Charles Dubin questioned Canadian sport: “have we, as Canadians, lost track of what athletic competitions is all about? Is there too much emphasis on winning the gold medal in Olympic Competition as the only achievement worthy of recognition?” (Dubin, 1990, p. 537). This commission led to subsequent changes in Canadian sport, including a stated shift in Canadian Sport policy to include foci on sport development and participation (Donnelly, 2013), however, funding for high-performance sport remains dependent upon performance outcomes and winning medals on the international stage (Kikulis, 2013). The current study provides further evidence that the focus and priority at the national team level is exclusively results-oriented.

A culture of fear and silence, and a lack of trust in sport organizations, particularly around the handling of safe sport issues, were commonly discussed as institutional barriers by athletes. Athletes reported a fear of repercussions for reporting and/or did not believe reports would be handled effectively, if at all, and that there were rarely consequences for the abusers. These findings echoed previous reports that athletes fear they won't be believed, and that sport organizations are likely to favor the coaches' reputations over the athlete's safety (Barrett, 2021). For example, in a testimony against Larry Nassar, Olympic Champion Simone Biles acknowledged the role sport organizations played in the sexual abuse she and over 300 other athletes experienced when declaring: “I blame Larry Nassar, and I also blame an entire system that enabled and perpetrated his abuse” (Barrett, 2021). In line with this sentiment, sport administrators have also reportedly minimized and trivialized reports of abuse from victims, blamed the victims for the abuse they experienced, and challenged the credibility of an athlete to defend the abuse (Parent, 2011).

The hesitations expressed by athletes regarding reporting align with Nite and Nauright's (2020) findings of case-handling of three prominent United States university sexual abuse cases. The investigations at each university were obscured by administrators' failures to investigate, inadequate investigations that were slow and kept quiet, a lack of appropriate action when a legal duty to report was necessary, and collusion between administrators and other stakeholders to minimize impacts (e.g., hidden police reports, forced silencing of board members). Additional concerns included the silencing of victims (e.g., persuaded not to report, bullied by fans and sport stakeholders to support the coach, shamed because they were “lucky” to have successful coaches) (Nite and Nauright, 2020). The findings of the current study reinforce those of previous research and athletes' fears that sport organizations are not sufficiently accountable when dealing with reports of maltreatment in sport. Moreover, they highlight a need to improve the transparency, fairness, and accountability of complaint and investigation processes.

Informed by the athletes' responses and previous research, recommendations are made for an independent third party to receive, investigate, and adjudicate concerns. More specifically, athletes need accessible, safe, and confidential avenues to raise their concerns, thus minimizing the overarching culture of fear and silence that persists in sport. The importance of this recommendation has been advocated in the sport literature (Donnelly and Kerr, 2018; Kerr and Kerr, 2020; Kerr et al., 2020) and by sport practitioners (Gurgis and Kerr, 2021). Third-party mechanisms can be beneficial because of their independence from the involved parties—avoiding self-governance and conflicts of interest—and can uniformly uphold standards across multiple sport organizations (Gurgis and Kerr, 2021). The establishment of third-party mechanisms is still in their infancy. Presently Canada is in the initial stages of implementing a national independent mechanism, although this will be accessible to national level athletes only (Sport Dispute Resolution Center of Canada, 2020).

The participants in the current study also recognized the importance of creating safe sport environments for all participants, and equity-deserving groups (e.g., LGBTQ2I+, women, parasport, and racialized athletes) in particular. Given previous research findings that equity-deserving athletes experience more violence of various forms (e.g., U. S. Center for Safe Sport, 2020; Willson et al., 2021), equity and inclusion need to be essential considerations in advancing safe sport. Of particular interest, athletes identified the need for equity and diversity in coaching and other positions of leadership, which has also been noted previously (Cunningham, 2008; Rankin-Wright et al., 2016; Sotiriadou et al., 2017; Joseph et al., 2021). Increasing diversity in leadership can be an essential part of creating a safe, welcoming, and inclusive environment by challenging the prominent sport norms that currently exist (e.g., racism, sexism) (Rankin-Wright et al., 2016; Sotiriadou et al., 2017; Joseph et al., 2021). The perspectives of athletes in the current study highlight the critical inclusion of equity, diversity, and inclusion within safe sport initiatives.

Education remains a prominent recommendation in the literature for all stakeholders for advancing safe sport and was echoed by the participants in this study. The participants in the current study highlighted education on power dynamics as a specific area of importance to advance safe sport. These notions are particularly relevant when considering that power differentials may place athletes in vulnerable situations where they are subjected to harmful experiences and the lack of attention currently given to power imbalances in safe sport education programs (Kerr et al., 2014).

Finally, athletes in this study recommended that a broader perspective of harm be adopted. Historically, in research and practice, the primary focus has been on sexual abuse, largely due to the media attention on such cases. However, there has been mounting evidence of other forms of harm occurring more often in sport; for instance, recent prevalence studies have indicated the prevalence of neglect, psychological and physical harm (Vertommen et al., 2016; Parent et al., 2019; Willson et al., 2021). Moreover, these forms of harm have been found to have equally deleterious outcomes as sexual harm, including decreased wellbeing, eating disorders, self-harm and suicide ideation (Willson et al., (in preparation)). The scope of harm should also be recognized outside the context of a coach-athlete relationship, particularly given evidence of perpetrators including peers, sport administrators, and team doctors (Vertommen et al., 2017; Willson et al., 2021). As highlighted by the athletes and previous research, there is a need to broaden the scope of attention on maltreatment to include all forms of harm.

A prominent theme across all barriers and suggestions is the misuse of power from those in positions of authority, including coaches, trainers, and sport administrators. The power imbalance between authority figures and athletes has left athletes vulnerable to abuse and unable to speak out about abuse they experienced. The lack of athlete power has been documented by researchers for decades, for instance, Kidd noted that Canadian athletes had become “workers of the state” because of their lack of agency in the sport structure (Kidd, 2013). Specifically, Kidd observed that Canadian elite athletes' financial compensation was bound to their contract, which also stipulated that they must comply with the rules and regulations of their sport organization (Kidd, 2013), thus compromising athlete agency and autonomy.

Bruyninckx critiqued the exceptionalism that sport organizations have, specifically the self-governance that allows sport to set and manage its own priorities, unchallenged by other public institutions (Bruyninckx, 2012). Autonomy and self-governance have enabled deliberative violence, racism, and discrimination to be treated as above the law because it occurred in a sport domain. The athletes' disclosures in the current study highlight concerns for this exceptionalism, particularly noting the autonomy and lack of fair process when it comes to maltreatment.

Many of the concerns expressed by the participants in this study reflect the growing recognition of the effects of structures of oppression in broader society. The MeToo Movement (Abrams and Bartlett, 2019) undoubtedly has influenced the willingness of athletes to speak of the sexual harms they have experienced and to pressure organizations for changes to the ways in which power is used. Similarly, the Black Lives Movement (Coombs and Cassilo, 2017) and the Truth and Reconciliation Commission in Canada (Rajwani et al., 2021), which have shone a light on structures of oppression and the high rates of violence experienced by Black and Indigenous individuals, have heightened attention on safety and inclusion within sport. In many ways, the recommendations athletes made in this study to enhance safety and inclusion in sport reflect broader challenges to power structures seen outside of sport.

### Future Directions

Several areas of interest emerge for future research. Most apparent is the need to include the athlete voice in research and in practice, including being a key contributor to decision-making practices. Currently in Canada, only 32 of the 49 National Sport Organizations have an established athlete representative on their board of directors, and the majority of these (62%) had only one reserved athlete position (AthletesCAN, 2020). More attention needs to be focused on enabling and supporting athletes to be actively engaged within these processes.

It would be beneficial to examine accountability and monitoring of sport organizations implementing safe sport initiatives (education, codes of conduct, independent safe sport mechanisms) through longitudinal case study research, including from athletes' perspectives. Such research is imperative to determine the transferability and effectiveness of Safe Sport initiatives in practice.

Additional research would benefit from qualitative approaches to elicit more in-depth responses from athletes, and to gain a more comprehensive understanding of athletes' subjective interpretations. Moreover, there is a need to expand the focus beyond National team athletes to include the full continuum of sport participants' experiences, from grassroots and youth sport to masters' levels.

The participants in the current study highlighted a lack of attention to the experiences of athletes in equity-deserving groups. Specifically, given preliminary reports that certain groups are more vulnerable to harm in sport (e.g., women, LGBTQ2I+, racialized) (Vertommen et al., 2016; Willson et al., 2021), exploration of potential qualitative differences in the challenges and recommendations for safe sport perceived by these populations is needed. We acknowledge the limitations of our own research team, being primarily White, thus having an inadequate understanding of the experiences of equity-deserving groups. Future research must prioritize diversity within research teams and as key focus of study in safe sport.

## Conclusion

The participants in the current study reported numerous challenges to advancing safe sport such as the win-at-all-costs mentality, normalization of harm, lack of attention to equity, diversity and inclusion, culture of fear and silence, and a lack of trust in sport organizations to handle safe sport issues. To address these challenges and in the pursuit of safe sport, the participants highlighted several recommendations, including prioritizing holistic development, education for all stakeholders, improving accountability of sport organizations, concrete actions to advance equity, diversity and inclusion, prohibiting sexual relationships between athletes and those in positions of power, and mandating independent complaint processes and ensuring accountability. This study also highlighted the problems associated with the autonomy and self-regulation of sport.

This research extends the current safe sport literature by highlighting athletes' perspectives on the challenges and recommendations to advancing safe sport. Given that athletes are the “reason-d'etre” of sport, it is important that athletes have platforms to share their opinions of what is needed to create a safe environment for them. From an applied perspective, we encourage sport organizations to include athletes within decision-making processes regarding safe sport. Future research would benefit from further study on ways to incorporate athletes' voices and perspectives effectively.

## Data Availability Statement

The datasets presented in this article are not readily available because the sensitive nature of the data (disclosing abuse) we do not feel it is appropriate to share this data. Requests to access the datasets should be directed to erin.willson@mail.utoronto.ca.

## Ethics Statement

The studies involving human participants were reviewed and approved by University of Toronto. The patients/participants provided their written informed consent to participate in this study.

## Author Contributions

EW contributed to the conceptualization of the study, data collection, led the data analysis, and was the lead writer. GK contributed to the conceptualization of the study, conceptualization of the results, and contributed to writing. AB assisted with the data analysis and contributed to the writing. AS assisted with the conceptualization of the study. All authors contributed to the article and approved the submitted version.

## Conflict of Interest

The authors declare that the research was conducted in the absence of any commercial or financial relationships that could be construed as a potential conflict of interest.

## Publisher's Note

All claims expressed in this article are solely those of the authors and do not necessarily represent those of their affiliated organizations, or those of the publisher, the editors and the reviewers. Any product that may be evaluated in this article, or claim that may be made by its manufacturer, is not guaranteed or endorsed by the publisher.

## Footnotes

^1^Available online at: https://www.safesportinternational.com (cited December 12, 2021).

^2^The Sport Information Resource Centre. *Safe Sport*. Available online at: https://sirc.ca/safesport/ (cited December 12, 2021).

^3^The Sport Information Resource Centre. *Universal Code of Conduct to Prevent and Address Maltreatment in Sport*. Available online at: https://sirc.ca/wp-content/uploads/2020/01/UCCMS-v5.1-FINAL-Eng.pdf (cited December 12, 2021).

^4^Sport Dispute Resolution Centre of Canada. *Investigation Unit*. Available online at: https://abuse-free-sport.ca/investigation-unit-overview/ (cited December 12, 2021).
